# Carbon nanotube-based flexible high-speed circuits with sub-nanosecond stage delays

**DOI:** 10.1038/s41467-022-34621-x

**Published:** 2022-11-08

**Authors:** Guanhua Long, Wanlin Jin, Fan Xia, Yuru Wang, Tianshun Bai, Xingxing Chen, Xuelei Liang, Lian-Mao Peng, Youfan Hu

**Affiliations:** 1 Key Laboratory for the Physics and Chemistry of Nanodevices, School of Electronics and Center for Carbon-Based Electronics, Peking University, 100871 Beijing, China; 2 Academy for Advanced Interdisciplinary Studies, Peking University, 100871 Beijing, China

**Keywords:** Electronic devices, Carbon nanotubes and fullerenes

## Abstract

High-speed flexible circuits are required in flexible systems to realize real-time information analysis or to construct wireless communication modules for emerging applications. Here, we present scaled carbon nanotube-based thin film transistors (CNT-TFTs) with channel lengths down to 450 nm on 2-μm-thick parylene substrates, achieving state-of-the-art performances of high on-state current (187.6 μA μm^−1^) and large transconductance (123.3 μS μm^−1^). Scaling behavior analyses reveal that the enhanced performance introduced by scaling is attributed to channel resistance reduction while the contact resistance (180 ± 50 kΩ per tube) remains unchanged, which is comparable to that achieved in devices on rigid substrates, indicating great potential in ultimate scaled flexible CNT-TFTs with high performance comparable to their counterparts on rigid substrates where contact resistance dominates the performance. Five-stage flexible ring oscillators are built to benchmark the speed of scaled devices, demonstrating a 281 ps stage delay at a low supply voltage of 2.6 V.

## Abstract (web summary)

High-speed flexible circuits are essential in flexible systems for real-time information analysis and wireless communication. Here, flexible circuits are reported with a 281 ps stage delay based on scaled carbon nanotube thin film transistors.

## Introduction

Carbon nanotubes (CNTs) are competitive candidates for building next-generation integrated circuits^1–4^ as well as strong participants in unconventional electronic technologies such as flexible electronics^5–11^. Flexible sensing systems interfacing with human bodies have shown enormous potential in health care applications, including but not limited to biosignal monitoring^12–14^. In such systems, high-speed circuits are in high demand. For example, the operating speeds of analog-to-digital converters, which are building blocks in interface circuits, determine the signal conversion rates and thus the overall throughputs of sensing systems. In addition, wireless system operations, especially real-time wireless data transmissions, also call for high-speed circuits to achieve a reasonably sized system. This is because the footprint of a wireless system with far-field communication capability is mainly limited by the size of the antenna *L*_antenna_, which depends on the frequency of the carrier wave *f*_carrier_ as 1/*L*_antenna _∝ *f*_carrier_. CNT-based thin-film transistors (CNT-TFTs) show promise for building high-speed flexible circuits^9^ due to the excellent electrical and mechanical properties of CNTs along with their low-temperature process capability.

Previous studies have shown that CNT-based circuits fabricated on flexible substrates can realize operation frequencies approaching 20 MHz with stage delays above 1 nanosecond^5,6,10,15–20^. However, service gaps remain for the application purpose we mentioned above. For example, the presumed corresponding *L*_antenna_ must be several meters long to work at such a frequency. Scaling devices down is the most straightforward approach to promote speed, as has been demonstrated in previous investigations on rigid substrates^21,22^, where ring oscillators (ROs) based on CNT-TFTs with deep sub-μm channel lengths (*L*_ch_ < 300 nm) provided oscillation frequencies over 1 GHz^23^ (stage delays down to several picoseconds). Nevertheless, flexible CNT-TFTs with sub-μm *L*_ch_ that can achieve this potential in performance and speed have not been reported.

In this work, we present the scaling of flexible CNT-TFTs with *L*_ch_ down to 450 nm on 2-μm-thick parylene substrates. The downscaled devices exhibit high performances of a width-normalized on-state current of 187.6 μA μm^−1^, and a width-normalized transconductance of 123.3 μS μm^−1^. A similar scaling trend and comparable performance with their rigid counterparts have been observed in flexible CNT-TFTs. Analysis based on the Y function method discovered that scaling led to a decrease in the channel resistance, while the contact resistance remained unchanged, which was 180 ± 50 kΩ per tube, comparable to that of rigid counterparts^24^. Therefore, further scaled flexible CNT-TFTs, in which contact resistance dominates the whole device performance, are expected to offer performance as excellent as rigid ones, extending their superiority in ultimate scaling. We utilized CNT-TFTs to construct 5-stage ROs, which delivered an oscillation frequency of 356 MHz at a low supply voltage of 2.6 V, equivalent to a stage delay of 281 ps, thus being the first flexible ROs with sub-ns stage delays.

## Results

### Fabrication of flexible CNT-TFTs

**Fig. 1:**
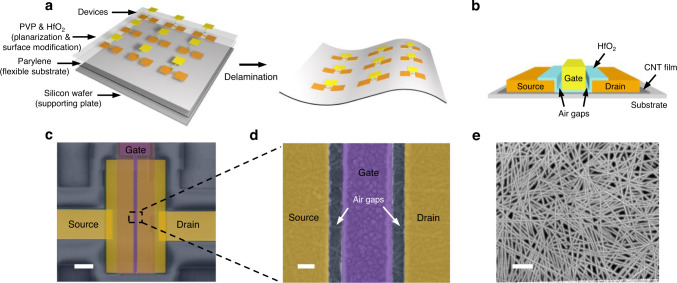
CNT-TFTs fabricated on a flexible parylene substrate. **a** Schematic illustration of device and circuit fabrication on a flexible parylene substrate. **b** Schematic diagram of a flexible CNT-TFT. **c** False-colored SEM image of a TFT with a channel length of 450 nm. Scale bar, 2 μm. **d** Magnified SEM image of the channel region of the device, scale bar, 100 nm. **e** SEM image of the randomly oriented CNT network in the channel. Scale bar, 200 nm.

Flexible CNT-TFTs are fabricated directly on an ultrathin parylene substrate (thickness ~2 μm) covered with a poly (4-vinyl-phenol) (PVP) layer for planarization and a 5-nm-thick HfO_2_ layer on the top to enhance surface adhesion for the subsequent device fabrication process (Supplementary Fig. 1). Figure 1a illustrates the overall construction of the system during fabrication. After device fabrication, the parylene foil was peeled off along with the devices by the previously reported capillary-assisted electrochemical delamination (CAED) method^25^. A schematic diagram of a flexible CNT-TFT is presented in Fig. 1b, in which a top-gate structure was adopted with a 10-nm-thick HfO_2_ dielectric layer to achieve a high gate control efficiency. Air gaps were introduced between the gate and source/drain electrodes to suppress the parasitic capacitance *C*. A thick Ti/Au (5 nm/120 nm) stack was applied as the gate electrode to minimize its resistance *R*, ensuring a small time constant *τ*_RC_ = *RC* toward high-speed circuits. Palladium (60 nm) was used to form the source and drain electrodes to selectively inject holes into CNT channel and control the polarity of the transistors to be p-type^26^. Moreover, a 20-nm-thick Au film was covered on the palladium to improve the mechanical robustness of the electrodes. Figure 1c shows a scanning electron microscopy (SEM) image of a CNT-TFT with a channel length *L*_ch_ = 450 nm. A magnified SEM image of the channel area is displayed in Fig. 1d to clearly show the nonoverlapping gate structure. A gate electrode with a gate length (*L*_g_) of 300 nm was positioned in the center of the channel with two 75-nm-long air gaps symmetrically distributed aside. Randomly oriented CNT networks with a relatively high density of ~60 tubes μm^−1^ in the channel are revealed by the SEM image shown in Fig. 1e. These CNTs with high semiconducting purity were sorted by using poly[9-(1-octylnonyl)−9H-carbazole-2,7-diyl] (PCz)^2^ and deposited on a parylene substrate by dip-coating, followed by a yttrium oxide coating and decoating (YOCD) process^27^ to remove the unwanted polymer residue.

### Characterization of flexible CNT-TFTs

**Fig. 2:**
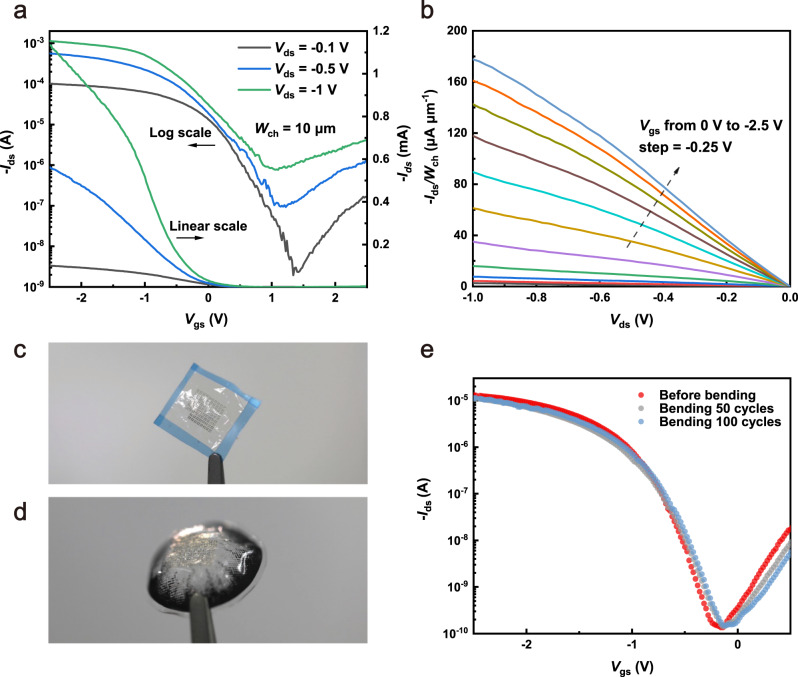
Characterization of flexible CNT-TFTs. **a** Transfer characteristics of a representative flexible CNT-TFT with *L*_ch_ = 450 nm under different *V*_ds_ biases. **b** Output characteristic of the representative CNT-TFT with *L*_ch_ = 450 nm. **c** Photograph of as-fabricated devices on a parylene substrate after the CAED process. **d** Photograph of the electronic foil attached to a contact lens. **e** Transfer characteristics of a CNT-TFT before and after the cyclic bending test.

The contact-controlled polarity and high semiconducting purity of CNTs enable the straightforward scaling down of devices on flexible substrates to achieve high speed. Transistors with different channel lengths (*L*_ch_ = 450 nm, 750 nm, 1 μm, 2 μm) were fabricated and characterized. Figure 2a displays the transfer characteristics (*I*_ds _– *V*_gs_) of a representative device with *L*_ch_ = 450 nm. Under a small drain-source voltage *V*_ds_ = −0.1 V, a large current on/off ratio (*I*_on_/*I*_off_) of 6 × 10^4^ and a subthreshold slope (SS) of ~140 mV dec^−1^ were obtained, suggesting that devices still possess outstanding gate control with a sub-μm-length channel. Such a high *I*_on_/*I*_off_ also confirms the high semiconducting purity of CNT materials. The output characteristics of the device, as shown in Fig. 2b, indicate that the Pd contacts are ohmic, and at *V*_ds_ = −1 V, the width-normalized on-state current (*I*_on_/*W*_ch_) reaches 177.7 μA μm^−1^. The relative permittivity (*ε*_r_) of HfO_2_ as gate dielectrics was measured to be 8, and the hole mobility *μ* was derived to be 64.2 cm^2^ s^−1^ V^−1^. The ultrathin, transparent electronic foil (Fig. 2c) shows good conformability, which can be attached to different curved surfaces, such as a contact lens (Fig. 2d), revealing the application potential of our devices in various scenarios such as health care monitoring. A cyclic bending test at a fixed radius of curvature of 1.3 cm was carried out (Supplementary Fig. 2). As shown in Fig. 2e, the transfer characteristics of the device (*L*_ch_ = 1 μm) were measured before and after 50 and 100 bending cycles. The subthreshold slope SS exhibits a slight decline from 165 mV dec^−1^ to 180 mV dec^−1^ after 100 bending cycles, while the threshold voltage and *I*_on_/*I*_off_ show no significant change during the bending experiment, confirming the good flexibility of our devices.

### Scaling behavior of flexible CNT-TFTs

**Fig. 3:**
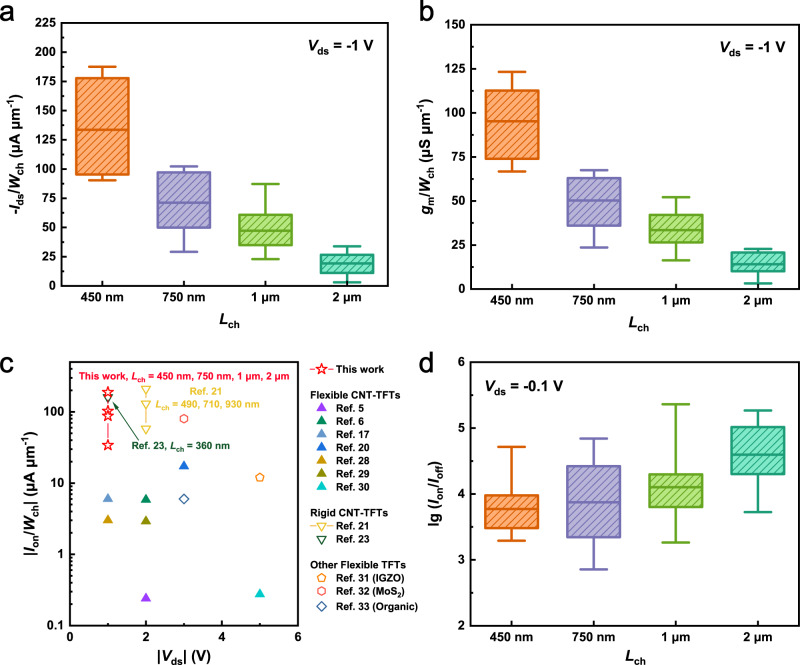
Scaling behavior of flexible CNT-TFTs. Statistical results of width-normalized **a** on-state current *I*_on_/*W*_ch_ and **b** transconductance *g*_m_/*W*_ch_ extracted from devices with *L*_ch_ = 450 nm, 750 nm, 1 μm, 2 μm. **c** *I*_on_/*W*_ch_ comparison of our flexible CNT-TFTs with other representative flexible TFTs and CNT-TFTs on rigid substrates. **d** Statistical results of the current on/off ratio log (*I*_on_/*I*_off_) from devices with different *L*_ch_. The upper, middle and bottom bars represent the maximum, average and minimum values, respectively. The upper and lower bands of the boxes represent the 75th and 25th percentiles.

Since the average length of a single carbon nanotube is ~1 μm, we chose *L*_ch_ = 450 nm, 750 nm, 1 μm, and 2 μm to study the scaling down behavior of CNT-TFT devices when the lengths of device channels are comparable with those of carbon nanotubes. The statistical results of *I*_on_/*W*_ch_ and width-normalized transconductance (*g*_m_/*W*_ch_) of these transistors are presented in Fig. 3a, b, and transfer characteristics of these devices are presented in Supplementary Fig. 3. It is revealed that shrinking the channel length toward the sub-μm region dramatically promotes the on-state performance, with the average values of *I*_on_/*W*_ch_ and *g*_m_/*W*_ch_ increasing from 18.23 ± 9.26 μA μm^−1^ and 14.10 ± 6.00 μS μm^−1^ to 133.60 ± 42.64 μA μm^−1^ and 95.23 ± 22.92 μS μm^−1^, respectively, as *L*_ch_ shrunk from 2 μm to 450 nm. During the downscaling process, device uniformity degradation was also observed as revealed from these two figures. The maximum *I*_on_/*W*_ch_ and *g*_m_/*W*_ch_ obtained from devices at *L*_ch_ = 450 nm are 187.6 μA μm^−1^ and 123.3 μS μm^−1^, respectively. We compared the on-state performance with existing flexible CNT-TFTs^5,6,17,18,20,28–30^, other flexible TFTs with *L*_ch_ > 200 nm^31–33^ and their CNT-based counterparts on rigid substrates^21,23^ recorded in the literature, as shown in Fig. 3c. The *I*_on_/*W*_ch_ value achieved in our device is the highest among these flexible devices and comparable with their counterparts on rigid substrates. A similar pattern of the scaling behaviors of CNT-TFTs with comparable *L*_ch_ on flexible and rigid substrates is also observed. This indicates the superiority of CNTs to serve in a flexible form without compromising electric performance even during scaling, revealing the considerable application potential of CNT-TFTs for building high-performance flexible ICs for high-speed applications. It is also notable that the voltage bias of −1 V is relatively small compared with other flexible TFTs, making our flexible CNT-TFTs promising in scenarios requiring low-voltage operations, such as implanted or epidermal electronic devices. The variation in *I*_on_/*I*_off_ in the downscaling process is also compared with the results shown in Fig. 3d. Although a slight decline is observed in *I*_on_/*I*_off_ from 4.60 ± 0.48 to 3.77 ± 0.45 decades as *L*_ch_ reduced from 2 μm to 450 nm, most devices with sub-μm channels still hold an *I*_on_/*I*_off_ > 10^3^. This should be attributed to the ultrahigh semiconducting purity of CNT materials and the established outstanding gate control efficiency in the device.

### Analysis of the scaling behavior via the Y function method

To rationalize the scaling down behavior of CNT-TFTs, the Y function method^24,34,35^ is used to analyze changes in the contact resistance 2*R*_*c*_ introduced by metal–tube contacts at the source/drain electrodes, the channel resistance *R*_ch_, and the total resistance *R*_total_ = 2*R*_*c*_ + *R*_ch_. The viability of the Y function method is based on two assumptions. First, the source–drain contacts are ohmic. In our case, the ohmic contact is confirmed by the data shown in Fig. 2b. Second, the carrier transportation is diffusive. For solution-processed single-walled CNTs, previous studies have shown that transport is diffusive even at an *L*_ch_ as small as 150 nm^35^.

**Fig. 4:**
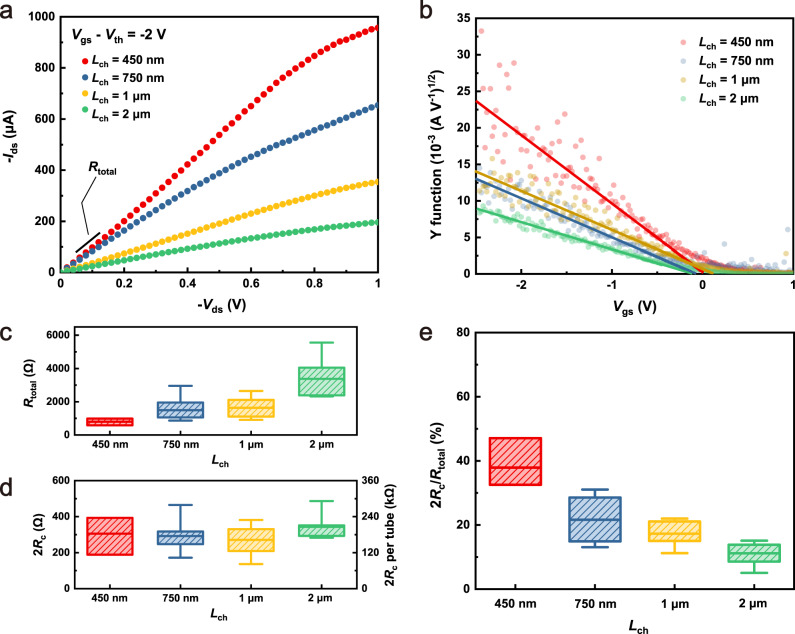
The Y function method analysis. **a** Output characteristics of representative TFTs with different *L*_ch_. *V*_gs_ – *V*_th_ is used in data analysis to minimize the influence of *V*_th_ variation. **b** Calculated Y function for the devices shown in **a** as a function of *V*_gs_. The extracted **c** device total resistance *R*_total_ and **d** contact resistance 2*R*_c_ of TFTs with different *L*_ch_. **e** The 2*R*_c_*/R*_total_ ratio of devices with different *L*_ch_ (*W*_ch_ = 10 μm). The upper, middle and bottom bars represent the maximum, average and minimum values, respectively. The upper and lower bands of the boxes represent the 75th and 25th percentiles.

The Y function of a transistor is defined as^36^

1 $$Y=\frac{{I}_{{{{\rm{ds}}}}}}{\sqrt{{g}_{{{{\rm{m}}}}}}}=\sqrt{{V}_{{{{\rm{ds}}}}}\cdot {G}_{{{{\rm{m}}}}}}({V}_{{{{\rm{gs}}}}}-{V}_{{{{\rm{th}}}}})$$

where *V*_th_ is the threshold voltage and *G*_m_ is the transconductance parameter defined as

2 $${G}_{{{{\rm{m}}}}}=\mu {C}_{{{{\rm{ox}}}}}\frac{{W}_{{{{\rm{ch}}}}}}{{L}_{{{{\rm{ch}}}}}}$$

where *μ* and *C*_ox_ stand for mobility and gate oxide capacitance, respectively. The output characteristics of representative transistors with different *L*_ch_ and the plots of the Y function versus *V*_gs_ are presented in Fig. 4a, b, respectively. The parameter *G*_m_ can be extracted from the slope of the linear region in Fig. 4b and is then further used to calculate 2*R*_c_ according to

3 $$2{R}_{{{{\rm{c}}}}}={R}_{{{{\rm{total}}}}}-{R}_{{{{\rm{ch}}}}}=\frac{{V}_{{{{\rm{ds}}}}}}{{I}_{{{{\rm{ds}}}}}}-\frac{1}{{G}_{{{{\rm{m}}}}}({V}_{{{{\rm{gs}}}}}-{V}_{{{{\rm{th}}}}})}$$

Figure 4c, d present the *R*_total_ obtained from the slope of the output characteristic curves in Fig. 4a in their linear region under small *V*_ds_ and the extracted 2*R*_c_, respectively. It is shown that *R*_total_ decreases with the shrinkage of the channel length, but 2*R*_*c*_ remains almost unchanged during the scaling process. This means that the performance improvement introduced by scaling should be ascribed mainly to the reduced *R*_ch_. The pattern of unchanged 2*R*_c_ during *L*_ch_ scaling falls in line with a previous CNT-TFT scaling study conducted on rigid substrates^24^, which had a comparable *L*_ch_ scaling range (from approximately twice to half the average length of a single CNT). Given the assumption that all metal-nanotube contacts are equal and connected in parallel, the 2*R*_c_ can be normalized on a single tube basis to quantify the average contact quality. In our case, with a CNT density of ~60 tubes μm^−1^ and a channel width of 10 μm, the 2*R*_c_ is estimated to be 180 ± 50 kΩ per tube (also shown in Fig. 3d), which is similar to the 2*R*_c_ value from the literature (136 ± 60 kΩ per tube) on rigid substrates^24^, indicating that working on flexible substrates brings no deterioration to device performance with respect to the contacts in our case. The distribution of 2*R*_c_ is also examined via transmission line method (TLM), with a similar value of 112 ± 12 kΩ per tube, as shown in Supplementary Fig. 4. Figure 4e shows the variation of the percentage 2*R*_c_/*R*_total_, which indicates that for devices with smaller *L*_ch_, the contact resistance contributed a greater proportion to the total resistance. It was previously revealed that 2*R*_c_ is the limiting factor (contributing most of *R*_total_) of the performance of the ultimate scaled CNT-TFTs that work in the quasi-ballistic regime^24,35^, where electron transport is mainly limited by the contacts. The ultimate scaled CNT-TFTs on rigid substrates have shown excellent electric performance (e.g., *I*_on_/*W*_ch_ ~270 μA μm^−1^ based on a CNT-network^37^, *I*_on_/*W*_ch_ = 1.92 mA μm^−1^ based on aligned CNT arrays^38^). It is prospective that the ultimate scaled flexible CNT-TFTs offer comparable on-state performance with rigid ones attributed to the similar scaling behavior and the achieved comparable 2*R*_c_, leaving considerable room for further performance boosting.

### Flexible inverters and ROs

**Fig. 5:**
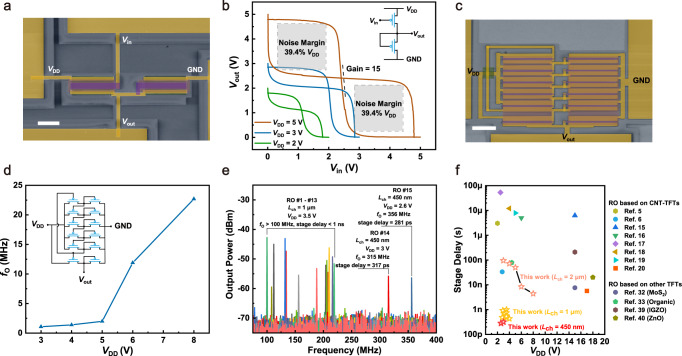
CNT-TFT-based flexible inverters and ROs. **a** SEM image of an inverter based on TFTs with *W*_ch_/*L*_ch_ = 10 μm/2 μm. Scale bar, 10 μm. **b** Voltage transfer characteristics and their mirrored curves of a representative inverter under different supply voltages Inset, circuit diagram of the inverter. **c** SEM image of a 5-stage RO based on TFTs with *W*_ch_/*L*_ch_ = 20 μm/1 μm. Scale bar, 10 μm. **d** Change in oscillation frequency *f*_O_ of the 5-stage RO based on TFTs with *L*_ch_ = 2 μm upon increasing supply voltage *V*_DD_. Inset, circuit diagram of the 5-stage RO. **e** Power spectra of multiple 5-stage ROs with *L*_ch_ of 1 μm and 450 nm, showing sub-ns stage delays. **f** Comparison of stage delays among representative flexible ROs.

Flexible inverters were fabricated and characterized with the aforementioned approach. Figure 5a shows an inverter constructed by transistors with *W*_ch_/*L*_ch_ = 10 μm/2 μm. The gate and source electrodes of the load transistor were connected to form a “zero-*V*_gs_” load (circuit diagram shown in the inset of Fig. 5b). The voltage transfer characteristics and their mirrored curves of a representative inverter under different supply voltages (*V*_DD_ = 2 V, 3 V, 5 V) are presented in Fig. 5b. The flexible inverter exhibits a near rail-to-rail output and the extracted voltage gain reaches approximately 15. The inverter also exhibits an excellent symmetric noise margin (NM) of 39.4% *V*_DD_ for both high and low NMs, possessing large noise tolerance. Five-stage ROs constructed of TFTs with *L*_ch_ = 2 μm, 1 μm and 450 nm were used to benchmark the speed of our devices (Fig. 5c and the inset of Fig. 5d). The correlation between oscillation frequency (*f*_O_) and operation voltage is plotted. As shown in Fig. 5d, the *f*_O_ of an RO constructed of TFTs with *L*_ch_ = 2 μm increases with increasing *V*_DD_ and reaches a maximum value of 22.73 MHz at *V*_DD_ = 8 V, which is equivalent to a stage delay of 4.4 ns. By downscaling *L*_ch_ of TFTs to 1 μm, stage delays of flexible ICs were pushed down to the sub-nanosecond region. As exhibited in Fig. 5e, *f*_O_ of multiple 5-stage ROs exceeded 100 MHz, giving sub-ns stage delays under *V*_DD_ = 3.5 V. The maximum *f*_O_ of 233 MHz, being equivalent to a stage delay of 430 ps, was obtained under *V*_DD_ = 4 V, with the waveform shown in Supplementary Fig. 5a. Sub-ns stage delays were also obtained from 9-stage and 15-stage ROs (Supplementary Fig. 5b), confirming the outstanding speed of devices. The speed of the RO was further elevated by scaling the *L*_ch_ down to 450 nm, in which a value of *f*_O_ = 356 MHz was achieved at *V*_DD_ = 2.6 V, as shown in Fig. 5e. This *f*_O_ result is equivalent to a stage delay of 281 ps. Compared with previous reports^5,6,15–20,32,33,39,40^ (as summarized in Fig. 5f), our RO is by far the fastest one achieved in a flexible form, based on any materials. The corresponding low supply voltage suggests the superiority of low power dissipation during continuous operation. Our results hint at the possibility of constructing flexible high-speed interface circuits, such as analog-to-digital converters with high sampling frequencies, for high-resolution processing of biosignals with small magnitudes. For wireless operation, with a frequency of 356 MHz reached, the size of the antenna can be shrunk to a reasonable length of <100 cm for integration. Therefore, the realization of such high-speed CNT-TFTs lays a solid foundation for building an integrated wireless sensing system.

In summary, we reported flexible CNT-TFTs with a short channel length down to 450 nm on parylene substrates. The downscaled flexible transistors presented a high electrical performance of *I*_on_/*W*_ch_ = 187.6 μA μm^−1^, *g*_m_/*W*_ch_ = 123.3 μS μm^−1^ and *I*_on_/*I*_off_ > 10^3^, which is comparable with those of rigid devices, confirming that the scaling capability of flexible CNT-TFTs is superior. Based on Y function analysis, during scaling, the *R*_ch_ of the device was reduced, while the 2*R*_c_, which is similar to that of rigid devices on a single tube basis, barely changed, suggesting that ultimate scaled flexible CNT-TFTs can achieve similar performance to their rigid counterparts. Shrinking devices led to a significant decrease in stage delays. Our 5-stage flexible ring oscillator demonstrated an oscillation frequency of 356 MHz, corresponding to a stage delay of 281 ps, which is the first flexible RO with a sub-ns stage delay. Furthermore, this high-speed operation was obtained under a low *V*_DD_ of 2.6 V. Our work demonstrated flexible circuits with both high speed and low power dissipation based on ultimate scaled flexible TFTs, which may lead to promising next-generation health care applications aided by flexible wireless systems.

## Methods

### Preparation of the parylene substrate

A silicon wafer was adopted as the supporting substrate during the fabrication process. A 2-μm-thick parylene-C film was deposited on the silicon wafer by chemical vapor deposition at room temperature (SCS Labcoter 2, Specialty Coating System). Then the solution of poly(4-vinyl-phenol) and poly-(melamine-co-formaldehyde) cross-linking agent mixed in propylene glycol monomethyl ether acetate (PGMEA) was spin-coated on the parylene-C film, followed by curing in vacuum for 2 h to form a planarization layer. Finally, a 5-nm-thick HfO_2_ film was deposited by atomic layer deposition (ALD) (TFS 200, Beneq Oy.) to enhance the adhesion between the metal electrodes and the flexible substrates.

### Preparation of the CNT thin film

One hundred milligrams of raw arc-discharged CNTs (Carbon Solutions Inc.) and 200 mg of 9-(1-octylnonyl)−9H-carbazole-2,7-diyl (PCz) were dispersed in 100 mL of chloroform with ultrasonication using a top-tip dispergator (VC500, Sonics) for 30 min at 300 W. Next, the solution was subjected to high-speed centrifugation at 20,000 × *g* for 1 h (Allegra X-22R, Beckman Coulter) for the removal of most of the bundles and insoluble materials. The upper supernatant was collected for a subsequent centrifugation at 50,000 × *g* for 2 h to further remove trace metallic nanotube contents. Finally, the upper 90% of the supernatant was collected as the CNT solution for use. The CNT thin film was fabricated via a dip-coating machine. First, the as-prepared parylene substrate with a silicon wafer as supporting plate was fixed on the dip-coating machine and vertically hung above the surface of the CNT solution. Then, the substrate was lowered into and withdrawn from the CNT solution at a controlled speed. As the solvent of the CNT solution evaporated at a high speed during the withdrawing process, the CNTs were left behind and coated on the surface of the substrate. This process was repeated 40 times to form the thin film of randomly oriented CNT network with a relatively high density (~60 tubes per μm).

### Yttrium oxide coating and decoating (YOCD) process

After the deposition of CNT thin film, a 3-nm-thick yttrium layer was deposited by electron-beam evaporation (DE 400, DE Technology). The wafer was then baked on a hot plate at 115 °C for 6 h for thorough oxidation of the yttrium film, and then a 1:10 diluted hydrochloride acid solution was used to remove the yttrium oxide. Finally, the wafer was rinsed with deionized water and dried by a nitrogen blow gun.

### Fabrication of flexible CNT-TFTs, inverters, and ring oscillators

As illustrated in Supplementary Fig. 6, electron-beam lithography (EBL) (Voyager, Raith GmbH.) was used to define the areas outside the channel region, which were followed by inductively coupled plasma reactive ion etching (Minilock, Trion) to remove the unwanted CNTs outside the channel region. After the removal, 60 nm palladium and 20 nm gold films were defined to form the source and drain electrodes by electron-beam lithography, electron-beam evaporation and a standard lift-off process. In ring oscillators, certain parts of interconnecting wires were later covered by an over-exposed PMMA bridge as the jumper, formed by an EBL-overexposure. A 10-nm-thick HfO_2_ film was later defined and deposited as the gate insulator by ALD. Finally, 5-nm-thick titanium and 120-nm-thick gold films were defined and deposited to form the gate electrodes.

### Characterization of CNT-TFTs, inverters, and ring oscillators

The electrical performances of the devices were measured using a semiconductor parameter analyzer (Keithley 4200A-SCS, Tektronix), an oscilloscope (DSO90404A, Agilent Technologies), a spectrum analyzer (N9020A, Agilent Technologies) and a probe station (Summit 1100, Cascade). All measurements were carried out at room temperature and in ambient air.

## Supplementary information

Supplementary Information

## Data Availability

The data that support the findings of this study are available within the paper and Supplementary Information. Additional relevant data are available from the corresponding authors upon reasonable request.
