# Supplementary material for: Carbon nanotube-based flexible high-speed circuits with sub-nanosecond stage delays
Source: Nat Commun. 2022 Nov 8;13:6734. doi: 10.1038/s41467-022-34621-x (PMC9643494; doi:10.1038/s41467-022-34621-x)
Supplement: Supplementary file 1 — Supplementary Information [file 41467_2022_34621_MOESM1_ESM.pdf]

# Carbon nanotube-based flexible high-speed circuits with sub-nanosecond stage delays

Guanhua Long[1#], Wanlin Jin[1#], Fan Xia[1,2], Yuru Wang[1], Tianshun Bai[1], Xingxing Chen[1], Xuelei Liang[1], Lian-Mao Peng[1,2]*, Youfan Hu[1,2]*

[1]Key Laboratory for the Physics and Chemistry of Nanodevices, School of Electronics and Center for Carbon-Based Electronics, Peking University, Beijing 100871, China

[2]Academy for Advanced Interdisciplinary Studies, Peking University, Beijing 100871, China

[#]Authors contributed equally to this work

*To whom correspondence should be addressed, Email: lmpeng@pku.edu.cn, youfanhu@pku.edu.cn

## Supplementary Information

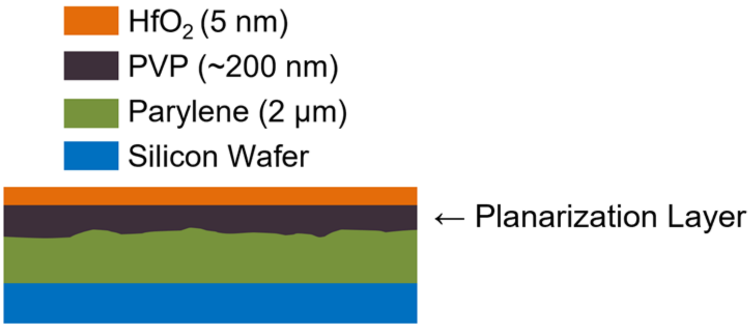


**Supplemetary Fig. 1: Structural illustration of the layered flexible substrate with silicon wafer as supporting plate during fabrication.**

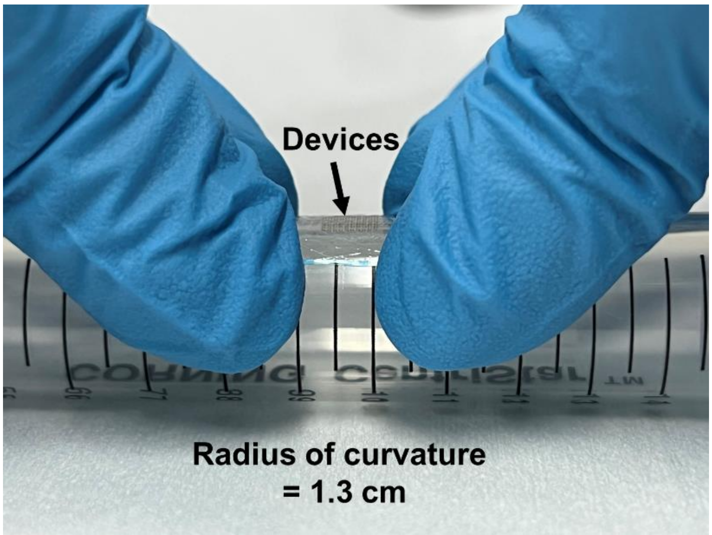


**Supplementary Fig. 2: Cyclic bending test carried out on a centrifuge tube with a radius of 1.3 cm.**

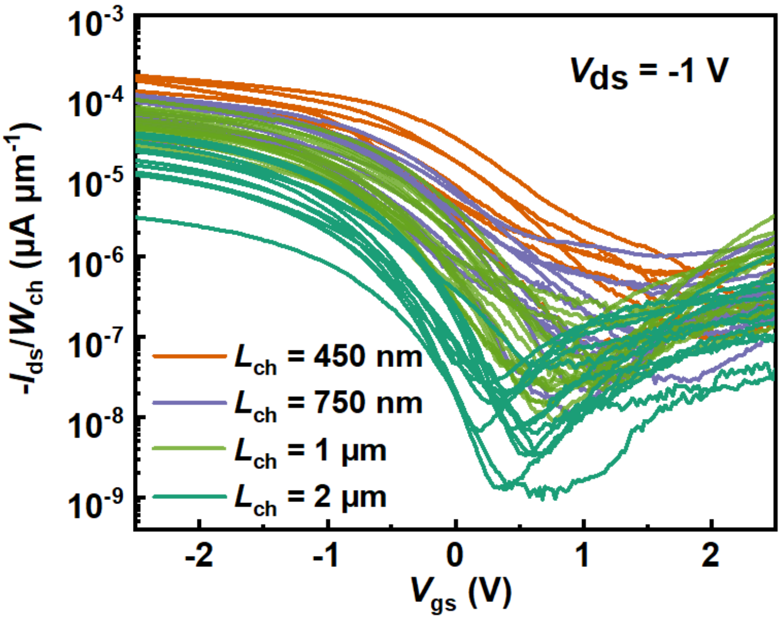


**Supplementary Fig. 3: Transfer characteristics curves of fabricated devices with different channel lengths.**

**Contact resistance analysis via transmission line method (TLM)**

To confirm the $2R_c$ results obtained via Y function method, a $2R_c$ analysis of the same devices based on transmission line method was also conducted. The $R_{total}$ of devices was modeled as $R_{total} = 2R_c + \rho_{ch}L_{ch}$, where $\rho_{ch}$ is the resistivity of channel material (in this case, the randomly oriented CNT film). With the assumption that $\rho_{ch}$ is constant during scaling, the $2R_c$ can be extracted from the y-intercept of the linear fitting of the $R_{total}$ versus $L_{ch}$ plot. Figure S2a shows the $R_{total}$ of each device (under $V_{gs}$-$V_{th}$ = -2.4 V) as a function of $L_{ch}$. The extracted $2R_c$ per device is 169 Ω, equivalent to 101 kΩ per tube on a single nanotube basis. Similar $2R_c$ of 185 Ω (111 kΩ per tube) and 206 Ω (124 kΩ per tube) were obtained under different $V_{gs}$-$V_{th}$ = -1.8 V and -2.1 V, respectively, as shown in Figure S2b. Our analysis via TLM provides a $2R_c$ result of 112 ± 12 kΩ per tube, which is consistent with our $2R_c$ result (180 ± 50 kΩ per tube) via the Y function method.

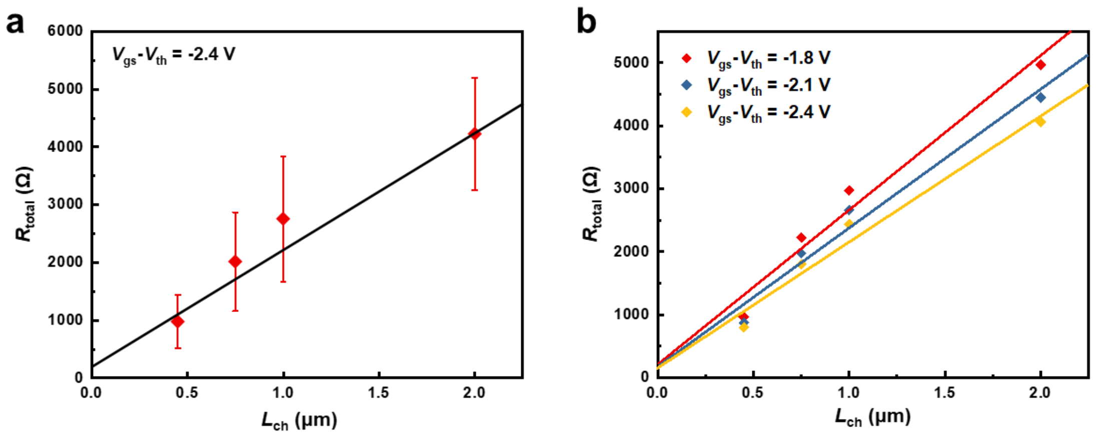


**Supplementary Fig. 4: Contact resistance analysis via TLM a,** $R_{total}$ as a function of $L_{ch}$ under $V_{gs}$-$V_{th}$ = -2.4 V. The red rhombi represent the average values of devices of each size, and corresponding standard deviation are highlighted by error bars. The black line represents the linear fitting result. **b,** Average $R_{total}$ as a function of $L_{ch}$ under different $V_{gs}$-$V_{th}$. Solid lines represents linear fitting results.

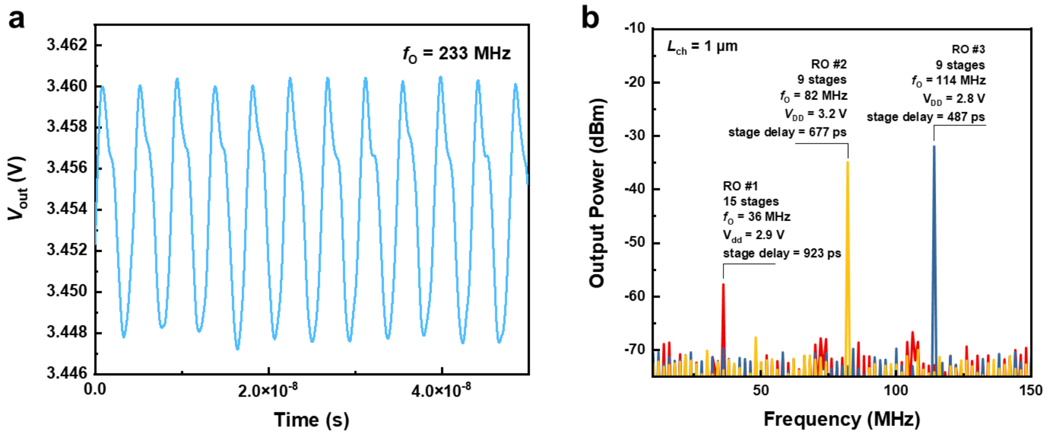


**Supplementary Fig. 5: Characterizations of flexible ring oscillators (ROs) with sub-ns stage delays. a,** Output waveform of a flexible 5-stage RO based on TFTs with $L_{ch}$ = 1 µm, showing an oscillation frequency of 233 MHz operated at $V_{DD}$ = 4 V. **b,** Power spectra of 9-stage and 15-stage ROs with $L_{ch}$ of 1 µm, showing stage delays below 1 ns.

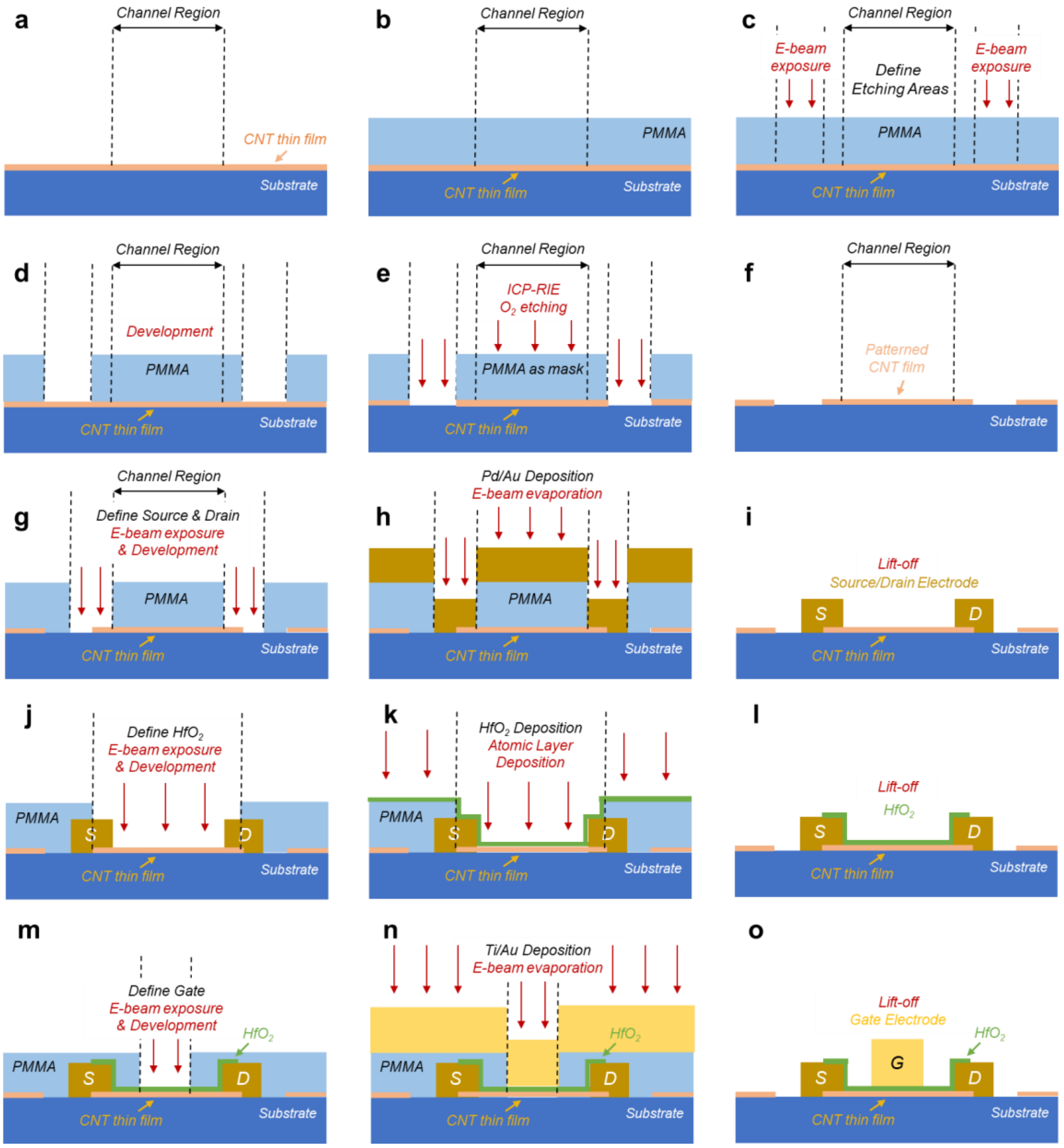


**Supplementary Fig. 6: Schematic illustration of the fabrication process. a-f,** Removal of the CNTs outside the channel region. **g-i,** Deposition of source and drain electrodes. **j-l,** Deposition of gate dielectrics. **m-o,** Deposition of gate electrode.
